# Individual and household determinants of overweight and obesity among children and adolescents in China

**DOI:** 10.7189/jogh.16.04096

**Published:** 2026-04-17

**Authors:** Meiqiyang Xue, Yutong Jiang, Mingsheng Chen, Lei Si

**Affiliations:** 1 School of Health Policy and Management, Nanjing Medical University, Nanjing, China; 2 School of Health Sciences, Faculty of Health, Western Sydney University, Campbelltown, Australia; 3 Translational Health Research Institute, Western Sydney University, Penrith, Australia

## Abstract

**Background:**

Overweight and obesity among children and adolescents are a global concern. Research on the combined influence of individual and household factors on overweight and obesity among children and adolescents across China remains relatively limited, especially regarding parental social capital. This study aims to identify the prevalence of overweight and obesity among children and adolescents in China and explore related individual and household determinants.

**Methods:**

We retrieved data on 2431 individuals aged 7–18 years from the 2020 China Family Panel Studies. We used binary logistic regression to evaluate associations between overweight and obesity among children and adolescents and 22 variables selected via keyword co-occurrence visualisation, and conducted sensitivity analyses to assess quality and robustness.

**Results:**

Among the participants, 20.36% (95% confidence interval (CI) = 18.78–22.02) were overweight, and 10.00% (95% CI = 8.83–11.26) were obese. Shared risk factors for both overweight and obesity included younger age, male sex, urban residence, informal education, paternal overweight, paternal obesity, maternal overweight, maternal loneliness, and low maternal cognitive social capital. Factors uniquely associated with overweight were maternal obesity, internet use, higher maternal structural social capital, good community infrastructure, and living in Northeastern China. For obesity, specific predictors were a larger mother-child age gap and the highest household income.

**Conclusions:**

Overweight and obesity among children and adolescents in China is shaped by complex individual and household factors. Our findings highlight the need for coordinated interventions by families, schools, and governments targeting intergenerational and sociocultural pathways.

Overweight and obesity among children and adolescents are linked to various diseases, including type II diabetes mellitus, metabolic syndrome, hypertension, cardiovascular diseases, and mental disorders [1]. Concerningly, the global age-standardised prevalence for obesity in school-aged children and adolescents increased substantially from 1990 to 2022, rising from 1.7% to 6.9% for girls and from 2.1% to 9.3% for boys [1]. This surge has been noted in 186 countries for the former and 195 countries for the latter population [1].

China has mirrored this global trend, but with an even steeper increase. The ongoing economic development and improving living standards have led to various lifestyle and environmental changes, including increased mechanised transportation, more sedentary lifestyles, and a dietary shift toward high-calorie, processed foods, contributing to the continued rise in the prevalence of overweight and obesity among children and adolescents [2]. Some studies have reported that the prevalence of overweight and obesity among Chinese children and adolescents aged 7–18 years has continuously increased from 1.2% in 1985 to 23.4% in 2019, marking more than a 19-fold increase, while the prevalence of obesity in particular rose from 0.1% in 1985 to 9.6% in 2019, representing a 96-fold increase [3].

Identifying factors associated with overweight and obesity among children and adolescents is crucial for implementing targeted treatments. However, existing studies have predominantly focused on its relationship with individual lifestyle factors such as nutritional intake, dietary habits, physical activity, and sleep duration [4]. Yet while effective lifestyle interventions that build on this understanding could help prevent overweight and obesity among children and adolescents in China [5], a comprehensive understanding of overweight and obesity among children and adolescents requires attention to broader contexts. Overweight and obesity among children and adolescents are not merely a personal health issue, but rather also a family-level concern. Few studies have examined the effects of household factors on overweight and obesity among children and adolescents in China, as they have largely explored the association between parental physical health and overweight and obesity among children and adolescents [4,6,7], often overlooking the impact of parental mental health, especially the role of parental social capital. Unlike individual physical indicators, social capital represents the capacity to mobilise external resources, which is essential for promoting healthy lifestyles, encouraging health-enhancing behaviours, and mitigating health-risk behaviours. Although previous studies have examined the link between personal social capital and health [8,9], its intergenerational transmission remains largely underexplored. By incorporating this dimension, our study shifts the focus from individual traits to the intergenerational pathways within families.

Given its escalating prevalence and the related severe health risks, it is imperative to prioritise overweight and obesity among children and adolescents as a public health concern. Understanding the magnitude of this issue and its determinants is essential for developing targeted interventions and informing policymaking. Therefore, we aimed to use a nationwide comprehensive data set (China Family Panel Studies, CFPS) to provide evidence on the prevalence and determinants of overweight and obesity among children and adolescents in China [10], with a focus on individual and household factors. We offer insights to support more effective prevention and control strategies for the overweight and obesity issues among children and adolescents in China.

## METHODS

### Data source and sampling method

We obtained data from the 2020 CFPS conducted by the Institute of Social Science Survey at Peking University. The CFPS is a nationwide representative longitudinal study that applies community, household, personal, and children’s questionnaires across 25 provinces, municipalities, and autonomous regions, representing 94.5% of the total population of mainland China. It uses a multi-stage probability sampling design with implicit stratification to retrieve a nationally representative sample from three strata: counties/districts, villages/resident committees, and households.

### Participants

We selected children and adolescents aged 7–18 years gathered through the personal, child, and household questionnaires in the 2020 CFPS. This age range is representative of children and adolescents in China and is widely used in health research and policy documents [3,11,12].

**Figure 1:**
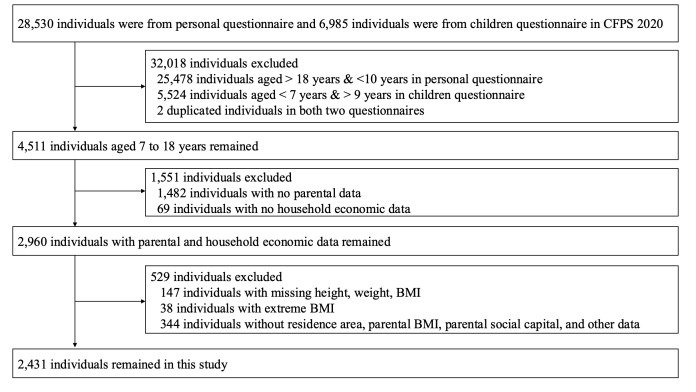
Flowchart of participant selection. Outliers were identified using the IQR method, where cases that exceeded 1.5 times the IQR above the third quartile or below the first quartile were excluded.

Specifically, the 2020 CFPS administered the personal questionnaire to 28 530 and the child questionnaire to 6985 individuals. After merging the data sets from the personal and child questionnaiers and removing duplicates, we excluded 31 002 individuals who were aged over 18 years and under 7 years. We also excluded 2 individuals who appeared in both questionnaires, leaving 4511 eligible individuals. When merging the household questionnaire, we further excluded 1482 individuals without parental information and 69 individuals without household economic data. We retained 2960 individuals after integrating parental and household data and removing invalid entries. Lastly, we excluded 529 individuals with extreme [13] or missing values for height, weight, body mass index (BMI), parental BMI, and household economic data. Finally, we included a total of 2431 individuals (**Figure 1**).

### Selection and grouping of independent variables

To identify the independent variables for this study and avoid an overly broad scope, we created a visualisation map of the co-occurrence frequency of related keywords using VOSviewer, version 1.6.20 (Centre for Science and Technology Studies, Leiden University, The Netherlands). Keywords associated with overweight and obesity among children and adolescents predominantly focused on individual factors, such as nutrition, sleep duration, and health consequences, such as hypertension, insulin-resistance, cardiovascular diseases, and depression. As research on family-related factors, especially parental social capital, was limited (Figure S1 in the **Online Supplementary Document**), we prioritised examining variables related to household factors to address research gaps, while also including individual variables for a comprehensive exploration of overweight and obesity among children and adolescents.

Based on the visualisation results, we selected 22 independent variables at the individual and household levels (**Table 1**). At the individual level, the independent variables included age, sex, residence area, informal education (referring to after-school programs aimed at enhancing academic performance or developing extracurricular talents), and internet use. At the household level, these included parental overweight and obesity status, parental education level, parental marital status, mother-child age gap, parental loneliness, parental cognitive social capital (individual subjective perceptions of trust and reciprocity norms in social networks) [14,15], parental structural social capital (formal or informal opportunities for building and participating in social networks) [16], household equivalised disposable income quantile, community infrastructure status (availability of education, healthcare, transportation, and other public facilities around the community), and household economic region (set in China to reflect regional socioeconomic development).

**Table 1:** Definition and grouping of 22 independent variables at the individual and household levels drawn from the CFPS 2020

	Definition
**Individual level**	
Age	A continuous variable ranging from 7 to 18 years
Sex	Classified as ‘Male’, ‘Female’
Residence area	Classified as ‘Urban’, ‘Rural’ according to the classification standard made by the Chinese National Bureau of Statistics
Informal education	Classified as ‘Yes’ or ‘No’
Internet use	Using mobile devices such as phones, tablets, and personal computers for internet use, classified as ‘Yes’ or ‘No’
**Household level**	
Maternal overweight status	Classified as ‘Yes’ or ‘No’
Maternal obesity status	Classified as ‘Yes’ or ‘No’
Paternal overweight status	Classified as ‘Yes’ or ‘No’
Paternal obesity status	Classified as ‘Yes’ or ‘No’
Maternal education level	Classified as ‘Illiterate’, ‘Below junior high school’, or ‘Junior high school and above’
Paternal education level	Classified as ‘Illiterate’, ‘Below junior high school’, or ‘Junior high school and above’
Parental marital status	Classified as ‘Married’ or ‘Others (Divorced, Widowed, Remarried)’
Mother–child age gap	A continuous variable representing the age gap between the mother and their offspring
Paternal loneliness days in a week	Classified as ‘Almost never (less than a day)’, ‘Sometimes (1–2 days)’, ‘Often (3–4 days)’, and ‘Most of the time (5–7 days)’ to reflect fathers’ loneliness status
Maternal loneliness days in a week	Classified as ‘Almost never (less than a day)’, ‘Sometimes (1–2 days)’, ‘Often (3–4 days)’, and ‘Most of the time (5–7 days)’ to reflect mothers’ loneliness status
Paternal cognitive social capital	Measured by the indicator ‘general trust’ with response options categorised as ‘Most people are trustworthy’ and ‘The more caution, the better’ Classified as ‘High’ and ‘Low’ to reflect fathers’ trust levels. ‘High’ indicates a high cognitive social capital while ‘Low’ reflects a low cognitive social capital.
Maternal cognitive social capital	Measured by the indicator ‘general trust’ with response options categorised as ‘Most people are trustworthy’ and ‘The more caution, the better’ Classified as ‘High’ and ‘Low’ to reflect mothers’ trust levels. ‘High’ indicates a high cognitive social capital while ‘Low’ reflects a low cognitive social capital.
Paternal structural social capital	Measured by the indicator ‘interpersonal relationships’ with the answer ranging from 0 to 10 to represent fathers’ interpersonal relationship strength, with higher values indicating more social connections and higher structural social capital.
Maternal structural social capital	Measured by the indicator ‘interpersonal relationships’ with the answer ranging from 0 to 10 to represent mothers’ interpersonal relationship strength, with higher values indicating more social connections and higher structural social capital.
Household equivalized disposable income	Classified into four groups: ‘lowest 25%’, ‘lower–middle 25%’, ‘upper–middle 25%’, and ‘highest 25%’ – based on the quartile distribution of household equivalised disposable income, which adjusts total household income by both household size and age composition to account for differences in economic resources *per capita*.
Community infrastructure status	Classified as ‘Good’, ‘Fair’, and ‘Poor’ based on respondents’ self-assessments. In rural areas, ‘community’ refers to a village, whereas in urban areas, it denotes a residential neighbourhood.
Household economic region	Classified as ‘Eastern region’, ‘Central region’, ‘Western region’, and ‘Northeastern region’ according to the classification standard made by the Chinese Bureau of Statistics.

### Definition and grouping of dependent variables

Overweight and obesity are typically defined as having an excess of body weight caused by an imbalance in calorie intake and expenditure, with affected individuals usually categorised by their BMI [17].

Here, we used the classification criteria developed by the Group of China Obesity Task Force to determine whether children and adolescents of different sexes or ages were overweight or obese [12] (Table S1 in the **Online Supplementary Document**). We chose this classification criterion because it closely aligns with international criteria, while also capturing the racial characteristics specific to the East Asian population, particularly the Chinese demographic. This criterion holds both practical significance and reflects a distinctively Chinese context [12].

### Statistical analysis

We first performed a descriptive analysis of independent and dependent variables to describe the prevalence of overweight and obesity among children and adolescents and the sociodemographic characteristics of children and adolescents included in this study. We assessed the normality of continuous variables using the Shapiro-Wilk test; as none of them followed a normal distribution, we summarised them as medians (MDs) with interquartile ranges (IQRs) and compared their between-group differences using Mann-Whitney U tests. We summarised categorical variables as frequencies and percentages and compared them across groups using χ^2^ tests.

Second, we employed binary logistic regression analyses to examine the factors influencing overweight and obesity among children and adolescents, using the odds ratio (OR) as the analytical metric. We constructed two models to explore the impact of variables at the individual and household levels on overweight and obesity among children and adolescents. Model 1 included variables from the individual level, while model 2 incorporated variables from both individual and household levels.

Finally, to ensure model appropriateness, we applied the rule of at least 10 outcome events per independent variable [18]. With 22 predictors, we required a minimum of 220 cases. Our sample included 243 participants with obesity and 495 overweight participants, providing enough events for reliable logistic regression. We evaluated model adequacy using receiver operating characteristic curves and corresponding area under the curve values, as well as with Hosmer–Lemeshow tests (Figure S2 and Table S2 in the **Online Supplementary Document**) [19]. Multicollinearity was not a concern, as all variance inflation factors were below 5 (Table S3 in the **Online Supplementary Document**). Additionally, interaction tests showed no significant effect between household income and economic region, suggesting that the association between income and overweight and obesity among children and adolescents was consistent across economic regions (Table S4 in the **Online Supplementary Document**). These results indicate that the statistical models were well-specified and appropriate for the analysis.

We performed data analysis using Stata, version 15.0 (StataCorp LLC, College Station, TX, USA). We set the statistical significance threshold at a two-tailed *P* < 0.05.

### Sensitivity analyses

We also performed sensitivity analyses to assess the robustness of the results for different model specifications. We replaced household equivalised disposable income with household *per capita* disposable income and explored its influence on the model outcomes. We adjusted the equivalised income for household size and composition, providing a better measure of individual economic well-being, while *per capita* income reflected the total income divided by household members, offering a simpler and more widely used metric. This substitution allowed us to test whether the choice of income measure impacts the results while holding all other model specifications constant. We also analysed the participants’ BMIs as continuous outcomes using a linear regression model instead of the binary overweight/obesity classification. This approach avoided reliance on specific thresholds and provided a complementary robustness check.

## RESULTS

### Descriptive analysis

Among 2431 individuals aged 7–18 years, 20.36% (95% confidence interval (CI) = 18.78–22.02) were overweight, while 10.00% (95% CI = 8.83–11.26) were obese. Descriptive analysis presented the detailed sociodemographic characteristics of the study participants (**Table 2**). The mean age of participants was 12.01 years, with 46.32% being male and 53.68% female. Of the participants, 45.87% resided in urban areas and 54.13% in rural areas. Participants were distributed across four regions of China. In addition, 36.82% of mothers and 50.19% of fathers were overweight. Key explanatory variables also included parental social capital, household income, and community infrastructure characteristics.

**Table 2:** Descriptive analysis of independent and dependent variables (n = 2431)*

Variable	Not overweight (n = 1936)	Overweight (n = 495)	*P*-value	Not obese (n = 2188)	Obese (n = 243)	*P*-value
Age, MD (IQR)	12 (6)	10 (5)	<0.001	12 (6)	9 (3)	<0.001
Sex						
*Male*	960 (73.56)	345 (26.44)	<0.001	1124 (86.13)	181 (13.87)	<0.001
*Female*	976 (86.68)	150 (13.32)		1064 (94.49)	62 (5.51)	
Residence area						
*Urban*	850 (76.23)	265 (23.77)	<0.001	985 (88.34)	130 (11.66)	<0.001
*Rural*	1086 (82.52)	230 (17.48)		1203 (91.41)	113 (8.59)	
Informal education						
*Yes*	467 (72.97)	173 (27.03)	<0.001	553 (86.41)	87 (13.59)	<0.001
*No*	1469 (82.02)	322 (17.98)		1635 (91.29)	156 (8.71)	
Internet use						
*Yes*	1253 (82.76)	261 (17.24)	<0.001	1403 (92.67)	111 (7.33)	<0.001
*No*	683 (74.48)	234 (25.52)		785 (85.61)	132 (14.39)	
Maternal overweight status						
*Yes*	673 (75.20)	222 (24.80)	<0.001	786 (87.82)	109 (12.18)	0.006
*No*	1263 (82.23)	273 (17.77)		1402 (91.28)	134 (8.72)	
Maternal obesity status						
*Yes*	110 (66.67)	55 (33.33)	<0.001	138 (83.64)	27 (16.36)	0.005
*No*	1826 (80.58)	440 (19.42)		2050 (90.47)	216 (9.53)	
Paternal overweight status						
*Yes*	913 (74.84)	307 (25.16)	<0.001	1066 (87.38)	154 (12.62)	<0.001
*No*	1023 (84.48)	188 (15.52)		1122 (92.65)	89 (7.35)	
Paternal obesity status						
*Yes*	206 (69.59)	90 (30.41)	<0.001	246 (83.11)	50 (16.89)	<0.001
*No*	1730 (81.03)	405 (18.97)		1942 (90.96)	193 (9.04)	
Maternal education level						
*Illiterate*	225 (81.82)	50 (18.18)	0.008	248 (90.18)	27 (9.82)	0.159
*Below junior high school*	404 (84.17)	76 (15.83)		443 (92.29)	37 (7.71)	
*Junior high school and above*	1307 (77.98)	369 (22.02)		1497 (89.32)	179 (10.68)	
Paternal education level						
*Illiterate*	112 (81.16)	26 (18.84)	0.030	122 (88.41)	16 (11.59)	0.089
*Below junior high school*	382 (83.96)	73 (16.04)		422 (92.75)	33 (7.25)	
*Junior high school and above*	1442 (78.45)	396 (21.55)		1644 (89.45)	194 (10.55)	
Parents’ marital status						
*Married*	1911 (79.53)	492 (20.47)	0.202	2162 (89.97)	241 (10.03)	0.613
*Other (divorced, widowed, remarried)*	25 (89.29)	3 (10.71)		26 (92.86)	2 (7.14)	
Mother-child age gap, MD (IQR)	26 (7)	26 (6)	0.5134	26 (7)	25 (6)	0.224
Paternal loneliness days in a week						
*Almost never (less than a day)*	1228 (79.59)	315 (20.41)	0.391	1393 (90.28)	150 (9.72)	0.725
*Sometimes (1–2 d)*	562 (78.82)	151 (21.18)		635 (89.06)	78 (10.94)	
*Often (3–4 d)*	89 (80.91)	21 (19.09)		100 (90.91)	10 (9.09)	
*Most of the time (5–7 d)*	57 (87.69)	8 (12.31)		60 (92.31)	5 (7.69)	
Maternal loneliness days in a week						
*Almost never (less than a day)*	1205 (80.76)	287 (19.24)	0.297	1358 (91.02)	134 (8.98)	0.173
*Sometimes (1–2 d)*	612 (77.47)	178 (22.53)		696 (88.10)	94 (11.90)	
*Often (3–4 d)*	82 (78.85)	22 (21.15)		93 (89.42)	11 (10.58)	
*Most of the time (5–7 d)*	37 (82.22)	8 (17.78)		41 (91.11)	4 (8.89)	
Paternal cognitive social capital						
*High*	1138 (79.86)	287 (20.14)	0.747	1284 (90.11)	141 (9.89)	0.843
*Low*	798 (79.32)	208 (20.68)		904 (89.86)	102 (10.14)	
Maternal cognitive social capital						
*High*	1139 (81.13)	265 (18.87)	0.033	1281 (91.24)	123 (8.76)	0.018
*Low*	797 (77.60)	230 (22.40)		907 (88.32)	120 (11.68)	
Paternal structural social capital, MD (IQR)	7 (2)	7 (3)	0.137	7 (3)	7 (2)	0.920
						
						
Maternal structural social capital, MD (IQR)	7 (3)	7 (2)	0.019	7 (3)	7 (2)	0.100
Household equivalised disposable income						
*Lowest 25%*	495 (81.55)	112 (18.45)	0.170	546 (89.95)	61 (10.05)	0.942
*Lower-middle 25%*	495 (81.28)	114 (18.72)		552 (90.64)	57 (9.36)	
*Upper-middle 25%*	474 (78.48)	130 (21.52)		542 (89.74)	62 (10.26)	
*Highest 25%*	472 (77.25)	139 (22.75)		548 (89.69)	63 (10.31)	
Community infrastructure status						
*Good*	723 (77.83)	206 (22.17)	0.026	831 (89.45)	98 (10.55)	0.774
*Fair*	990 (79.77)	251 (20.23)		1121 (90.33)	120 (9.67)	
*Poor*	223 (85.44)	38 (14.56)		236 (90.42)	25 (9.58)	
Household economic region						
*Eastern region*	559 (78.29)	155 (21.71)	0.002	639 (89.50)	75 (10.50)	0.305
*Central region*	579 (80.64)	139 (19.36)		643 (89.55)	75 (10.45)	
*Western region*	656 (82.21)	142 (17.79)		730 (91.48)	68 (8.52)	
*Northeastern region*	142 (70.65)	59 (29.35)		176 (87.56)	25 (12.44)	

*Presented as n (%) unless specified otherwise.

IQR – interquartile range, MD – median

### Logistic regression analysis for children and adolescent overweight

Model 1 indicated that individual factors, including younger age (OR = 0.89; 95% CI = 0.86–0.92, *P* < 0.001), male sex (OR = 2.40; 95% CI = 1.93–2.98, *P* < 0.001), urban residence (OR = 1.37; 95% CI = 1.11–1.70, *P* = 0.003), and informal education (OR = 1.59; 95% CI = 1.27–1.99, *P* < 0.001), were significantly associated with overweight. These associations remained consistent in model 2, where internet use also emerged as negatively related to overweight (OR = 0.76; 95% CI = 0.59–0.98, *P* = 0.036). Household-level influences included paternal overweight (OR = 1.64; 95% CI = 1.29–2.07, *P* < 0.001) and obesity (OR = 1.44; 95% CI = 1.05–1.97, *P* = 0.024), maternal overweight (OR = 1.52; 95% CI = 1.20–1.93, *P* < 0.001) and obesity (OR = 1.51; 95% CI = 1.01–2.26, *P* = 0.043), slight maternal loneliness (OR = 1.26; 95% CI = 1.00–1.59, *P* = 0.047), lower maternal cognitive social capital (OR = 1.35; 95% CI = 1.09–1.68, *P* = 0.007), maternal structural social capital (OR = 1.08; 95% CI = 1.01–1.15, *P* = 0.017), good community infrastructure (OR = 0.60; 95% CI = 0.40–0.91, *P* = 0.015), and residence in Northeastern region (OR = 1.51; 95% CI = 1.02–2.24, *P* = 0.039) (**Table 3**).

**Table 3:** Logistic regression analysis for overweight and obesity among children and adolescents

	Overweight model 1		Overweight model 2		Obesity model 1		Obesity model 2	
	**OR (95% CI)**	***P*-value**	**OR (95% CI)**	***P*-value**	**OR (95% CI)**	***P*-value**	**OR (95% CI)**	***P*-value**
**Individual level**								
Age	0.89 (0.86–0.92)	<0.001	0.87 (0.84–0.91)	<0.001	0.76 (0.72–0.81)	<0.001	0.73 (0.69–0.78)	<0.001
Sex (ref: female)								
*Male*	2.40 (1.93–2.98)	<0.001	2.52 (2.01–3.15)	<0.001	2.84 (2.08–3.87)	<0.001	3.06 (2.22–4.21)	<0.001
Residence area (ref: rural)								
*Urban*	1.37 (1.11–1.70)	0.003	1.34 (1.06–1.69)	0.014	1.25 (0.94–1.67)	0.122	1.50 (1.09–2.06)	0.013
Informal education (ref: no)								
*Yes*	1.59 (1.27–1.99)	<0.001	1.50 (1.18–1.92)	0.001	1.50 (1.11–2.03)	0.009	1.58 (1.14–2.19)	0.006
Internet use (ref: no)								
*Yes*	0.80 (0.63–1.01)	0.062	0.76 (0.59–0.98)	0.036	0.91 (0.67–1.25)	0.578	0.97 (0.69–1.36)	0.859
**Household level**								
Paternal overweight status (ref: no)								
*Yes*			1.64 (1.29–2.07)	<0.001			1.65 (1.19–2.29)	0.002
Paternal obesity status (ref: no)								
*Yes*			1.44 (1.05–1.97)	0.024			1.68 (1.12–2.52)	0.013
Maternal overweight status (ref: no)								
*Yes*			1.52 (1.20–1.93)	<0.001			1.62 (1.17–2.23)	0.003
Maternal obesity status (ref: no)								
*Yes*			1.51 (1.01–2.26)	0.043			1.50 (0.88–2.56)	0.135
Paternal education level (ref: illiterate)								
*Below junior high school*			0.79 (0.45–1.39)	0.411			0.56 (0.26–1.18)	0.129
*Junior high school and above*			0.87 (0.50–1.52)	0.632			0.65 (0.31–1.38)	0.261
Maternal education level (ref: illiterate)								
*Below junior high school*			0.65 (0.41–1.04)	0.071			0.56 (0.29–1.06)	0.077
*Junior high school and above*			0.71 (0.45–1.13)	0.148			0.43 (0.23–0.82)	0.01
Parental marital status (ref: married)								
*Other*			0.50 (0.14–1.75)	0.277			0.91 (0.20–4.17)	0.899
Mother–child age gap			1.00 (0.98–1.02)	0.906			0.97 (0.94–1.00)	0.037
Paternal loneliness days in a week (ref: almost never (less than a day))								
*Sometimes (1–2 days)*			1.04 (0.82–1.32)	0.735			1.15 (0.83–1.58)	0.403
*Often (3–4 days)*			1.01 (0.59–1.72)	0.966			1.08 (0.52–2.24)	0.846
*Most of the time (5–7 days)*			0.50 (0.23–1.11)	0.087			0.67 (0.25–1.81)	0.427
Maternal loneliness days in a week (ref: almost never (less than a day))								
*Sometimes (1–2 days)*			1.26 (1.00–1.59)	0.047			1.38 (1.02–1.89)	0.039
*Often (3–4 days)*			1.29 (0.76–2.21)	0.342			1.12 (0.55–2.31)	0.753
*Most of the time (5–7 days)*			1.15 (0.50–2.64)	0.736			1.28 (0.42–3.92)	0.668
Maternal cognitive social capital (ref: high)								
*Low*			1.35 (1.09–1.68)	0.007			1.45 (1.08–1.95)	0.013
Paternal cognitive social capital (ref: high)								
*Low*			1.00 (0.80–1.25)	0.992			0.90 (0.67–1.22)	0.508
Maternal structural social capital			1.08 (1.01–1.15)	0.017			1.05 (0.96–1.14)	0.265
Paternal structural social capital			0.96 (0.91–1.03)	0.253			1.04 (0.96–1.13)	0.362
Household equivalized disposable income (ref: lowest 25%)								
*Lower–middle 25%*			0.98 (0.72–1.35)	0.916			0.94 (0.62–1.43)	0.770
*Upper–middle 25%*			1.05 (0.77–1.43)	0.771			0.89 (0.59–1.36)	0.605
*Highest 25%*			0.89 (0.63–1.24)	0.480			0.62 (0.39–0.98)	0.041
Community infrastructure status (ref: poor)								
*Fair*			0.93 (0.74–1.17)	0.543			0.96 (0.70–1.32)	0.795
*Good*			0.60 (0.40–0.91)	0.015			0.92 (0.55–1.54)	0.746
Household economic region (ref: Eastern region)								
*Central region*			0.86 (0.65–1.14)	0.294			0.96 (0.66–1.39)	0.827
*Western region*			0.86 (0.63–1.16)	0.317			0.69 (0.45–1.06)	0.090
*Northeastern region*			1.51 (1.02–2.24)	0.039			1.28 (0.74–2.21)	0.374

CI – confidence interval, OR – odds ratio

### Logistic regression results for children and adolescent obesity

For model 1, younger age (OR = 0.76; 95% CI = 0.72–0.81, *P* < 0.001), male sex (OR = 2.84; 95% CI = 2.08–3.87, *P* < 0.001), and informal education (OR = 1.50; 95% CI = 1.11–2.03, *P* = 0.009) were significantly linked with obesity. The relationships of individual factors remain consistent for model 2, with urban residence also being linked to children and adolescent obesity (OR = 1.50; 95% CI = 1.09–2.06, *P* = 0.013). Household-level influences included paternal overweight (OR = 1.65; 95% CI = 1.19–2.29, *P* = 0.002), maternal overweight (OR = 1.62; 95% CI = 1.17–2.23, *P* = 0.003), paternal obesity (OR = 1.68; 95% CI = 1.12–2.52, *P* = 0.013), mother-child age gap (OR = 0.97; 95% CI = 0.94–1.00, *P* = 0.037), slight maternal loneliness (OR = 1.38; 95% CI = 1.02–1.89, *P* = 0.039), lower maternal cognitive social capital (OR = 1.45; 95% CI = 1.08–1.95, *P* = 0.013), and belonging to the highest household income (OR = 0.62; 95% CI = 0.39–0.98, *P* = 0.041) (**Table 3**).

### Sensitivity analysis

When using equivalised disposable income to assess the impact of household economic status (Table S5 and S6 in the **Online Supplementary Document**), individuals from the highest income group were significantly less likely to be obese than those in the lowest income group (OR = 0.62; 95% CI = 0.39–0.98, *P* = 0.041). However, this association became statistically insignificant during the sensitivity analysis when equivalised disposable income was replaced by *per capita* household disposable income (OR = 0.65; 95% CI = 0.40–1.04, *P* = 0.070). Meanwhile, mothers reporting feeling lonely ‘sometimes’ in a week had a higher risk of having overweight offspring than those who responded ‘almost never’ to the same question (OR = 1.26; 95% CI = 1.00–1.59, *P* = 0.047). This association, however, also became non-significant in the above-mentioned sensitivity analysis (OR = 1.26; 95% CI = 1.00–1.58, *P* = 0.051). The significance and direction of other variables remained consistent across both models.

When treating BMI as a continuous outcome in linear regression models (Table S7 in the **Online Supplementary Document**), he associations between paternal overweight status (*β* = 0.76; 95% CI = 0.48–1.04, *P* < 0.001), paternal obesity status (*β* = 0.80; 95% CI = 0.38–1.23, *P* < 0.001), maternal overweight status (*β* = 0.88; 95% CI = 0.59–1.17, *P* < 0.001), and maternal obesity status (*β* = 0.85; 95% CI = 0.29–1.40, *P* = 0.003) and offspring’s BMI remained consistent with those observed in the logistic models. Similarly, maternal social capital showed comparable results to the primary analysis, indicating that individuals whose mothers had low cognitive social capital were more likely to have a higher BMI (*β* = 0.28; 95% CI = 0.01–0.55, *P* = 0.039). In contrast, the effects of internet use, community infrastructure, economic region, and household income were not statistically significant in the linear regression models.

### Comparative analysis of logistic regression results for overweight and obesity among children and adolescents

The logistic regression analyses comparing overweight and obesity among children and adolescents revealed both shared patterns and notable differences across associated factors. Specifically, several predictors were consistent across both outcomes: younger age (overweight model 2: OR = 0.87; 95% CI = 0.84–0.91, *P* < 0.001; obesity model 2: OR = 0.73; 95% CI = 0.69–0.78, *P* < 0.001), male sex (overweight model 2: OR = 2.52; 95% CI = 2.01–3.15, *P* < 0.001; obesity model 2: OR = 3.06; 95% CI = 2.22–4.21, *P* < 0.001), urban residence (overweight model 2: OR = 1.34; 95% CI = 1.06–1.69, *P* = 0.014; obesity model 2: OR = 1.50; 95% CI = 1.09–2.06, *P* = 0.013), informal education (overweight model 2: OR = 1.50; 95% CI = 1.18–1.92, *P* = 0.001; obesity model 2: OR = 1.58; 95% CI = 1.14–2.19, *P* = 0.006), paternal overweight (overweight model 2: OR = 1.64; 95% CI = 1.29–2.07, *P* < 0.001; obesity model 2: OR = 1.65; 95% CI = 1.19–2.29, *P* = 0.002), paternal obesity (overweight model 2: OR = 1.44; 95% CI = 1.05–1.97, *P* = 0.024; obesity model 2: OR = 1.68; 95% CI = 1.12–2.52, *P* = 0.013), maternal overweight (overweight model 2: OR = 1.52; 95% CI = 1.20–1.93, *P* < 0.001; obesity model 2: OR = 1.62; 95% CI = 1.17–2.23, *P* = 0.003), maternal loneliness (overweight model 2: OR = 1.26; 95% CI = 1.00–1.59, *P* = 0.047; obesity model 2: OR = 1.38; 95% CI = 1.02–1.89, *P* = 0.039), and lower maternal cognitive social capital (overweight model 2: OR = 1.35; 95% CI = 1.09–1.68, *P* = 0.007; obesity model 2: OR = 1.45; 95% CI = 1.08–1.95, *P* = 0.013) all increased the risk of overweight and obesity among children and adolescents. However, we observed some outcome-specific differences. Internet use (OR = 0.76; 95% CI = 0.59–0.98, *P* = 0.036), maternal obesity (OR = 1.51; 95% CI = 1.01–2.26, *P* = 0.043), higher maternal structural social capital (OR = 1.08; 95% CI = 1.01–1.15, *P* = 0.017), good community infrastructure (OR = 0.60; 95% CI = 0.40–0.91, *P* = 0.015), and living in the Northeastern economic region (OR = 1.51; 95% CI = 1.02–2.24, *P* = 0.039) were only linked to overweight, while a larger mother-child age gap (OR = 0.97; 95% CI = 0.94–1.00, *P* = 0.037) and highest household income (OR = 0.62; 95% CI = 0.39–0.98, *P* = 0.041) were uniquely associated with obesity.

## DISCUSSION

The results of our descriptive analysis showed the prevalence of overweight and obesity among children and adolescents in China to be severe, with 20.36% of children and adolescents being overweight and 10.00% being obese. Four main findings emerged. First, parental influences on overweight and obesity among children and adolescents exhibited a gender-asymmetric pattern: parental overweight and obesity were associated with overweight and obesity among children and adolescents’ risk through physiological pathways, while maternal psychosocial factors were significant predictors for both conditions. Second, the association between household income and obesity inversed, suggesting a shift in the socioeconomic patterning of children and adolescent obesity. Third, children and adolescents receiving informal education had higher odds of overweight and obesity among children and adolescents, highlighting the potential role of academic pressure. Fourth, we observed population-level disparities, with a higher prevalence of overweight and obesity among children and adolescents, particularly among males, younger individuals, and urban residents compared with their respective counterparts; additionally, adolescents residing in the Northeastern region were more likely to be overweight.

### Differences in parental influence: a gender-asymmetric mechanism

A key contribution of this study is the identification of paternal and maternal influence pathways for children and adolescents at risk of overweight and obesity, suggesting that parental factors play a gender-asymmetric complementary mechanism. The overweight/obesity status of parents both exhibited physiological effects, whereas maternal cognitive social capital and maternal loneliness were highlighted as psychosocial predictors. This dual emphasis highlights that both parents influence intergenerational overweight and obesity risks, though through partly different mechanisms. Specifically, we found that parental overweight and obesity were strongly associated with overweight and obesity among children and adolescents, which aligns with the results of prior research [20]. The association may be partly explained by genetic predisposition and metabolic factors, but also may be reinforced by shared family environments and health-related parenting behaviours, suggesting that parental biological inheritance and lifestyle contexts contribute to overweight and obesity among children and adolescents [20].

Furthermore, maternal cognitive social capital was inversely related to overweight and obesity among children and adolescents, suggesting that higher social trust and stronger social networks may facilitate healthier parenting behaviours and offspring’s health outcomes. Maternal loneliness was also linked to increased obesity risk. To our knowledge, no prior research has directly examined a link between parental social capital and overweight and obesity among children and adolescents. However, potential mechanisms can be drawn from related studies. First, evidence suggested that adults with lower cognitive social capital had a higher risk of obesity, whereby higher social capital facilitates the diffusion of health-promoting behaviours and provides access to diverse information regarding nutrition and healthy lifestyles [9]. The obesity risk may extend to the next generation through the household environment and the intergenerational transmission of social capital, as parental social capital shapes children and adolescents’ perception of sociability [21].

In the Chinese context, where mothers typically shoulder the majority of parenting responsibilities and children and adolescents often spend more time with mothers than fathers, maternal social capital may play a stronger role [22]; hence, mothers with low social capital are more likely to have offsprings with similarly low social capital. Moreover, mothers with poor mental health may adopt a permissive parenting style, characterised by reduced involvement in guiding and shaping children’s dietary choices and health behaviours, thereby increasing the risk of overweight and obesity [23]. Furthermore, research on children and adolescent social capital has shown that children and adolescents with lower social capital are more likely to have lower participation in physical activities [24], thereby leading to overweight and obesity.

Sex differences in the perception and expression of social trust may also contribute to these findings. Men and women interpret and express social trust differently, with the former being less likely to acknowledge loneliness or prioritise social trust due to cultural norms discouraging emotional expression [25,26]. This may lead to an underreporting of trust among fathers, thereby attenuating its observed impact on children's and adolescents' health. Maternal structural social capital does not have a similar effect, which aligns with the conclusion of Straughan et al [27] that parental social network support does not necessarily translate into better health for children. These findings indicate that quality, rather than quantity of maternal social relationships matters in the prevention of overweight and obesity among children and adolescents. Decisions-makers should thus emphasise the role of mother-child interaction when developing interventions and policies.

### Shifting socioeconomic patterns in obesity prevalence

We found that replacing equivalised disposable income with household *per capita* during the sensitivity analysis showed the association between household income and obesity among children and adolescentes to be insignificant. As equivalised disposable income accounts for household size and composition, it reflects individual economic resources more accurately. Considering population weighting, our results consistently show that higher income is linked to a lower risk of children and adolescent obesity.

Traditionally, higher household income has been associated with increased obesity in children and adolescents in China [28]. However, we found that households with the highest equivalised disposable income are now associated with a lower risk of obesity, suggesting that China may be transitioning toward the contrary pattern observed in high-income countries [29-31]. This shift may be attributed to greater health awareness and better access to nutritional knowledge and lifestyle choices among wealthier families, whereas lower-income families may face barriers to adopting healthier behaviours due to financial and environmental constraints. These results suggest that obesity prevention efforts should reassess the importance of socioeconomic factors and focus more on lower-income populations, where obesity risk may be rising due to economic disparities in health resources.

### Academic pressure and overweight and obesity among children and adolescents

We found that excessive academic pressure from after-school informal education is a key contributor to overweight and obesity among children and adolescents. Existing research has shown that when children and adolescents devote more time to academic learning, they directly spend more time on sedentary activities and correspondingly less time engaging in physical activities, which reduces energy expenditure [32–34]. Moreover, extended study time may be at the expense of adequate sleep, while sleep deprivation has been consistently linked to a higher risk of overweight and obesity among children and adolescents. These factors create a pathway through which academic pressure contributes to the development of overweight and obesity among children and adolescents. Therefore, it is crucial to reassess the significance of academic pressure when developing policies to address CAOO, rather than simply advocating for increased exercise time.

### Population-based disparities in overweight and obesity among children and adolescents

We noted a higher prevalence of overweight and obesity among male than female children and adolescents, a greater prevalence among younger children and adolescents, and a higher prevalence among urban children and adolescents than among their counterparts in rural areas. This is consistent with other studies conducted in China [3,35,36]. While there are some potential biological explanations for these findings, such as sex-related genetic expression and physiological characteristics, sociocultural factors also appear to play an important role. In Chinese society, being thin is often valued among girls, whereas a larger body size is viewed as a sign of strength and health among boys [37]. Parents are also more likely to select overweight/obesity as the ideal body image for boys than for girls, reflecting the very same cultural norms [37,38]. Such attitudes may lead parents to underestimate boys’ overweight status and show less concern for boys’ weight control, while girls’ weight is more closely monitored [37,39]. Urban residents benefit from better living conditions but engage in less physical activity than their rural counterparts, which contributes to overweight and obesity among children and adolescents [35,40]. Additionally, older children and adolescents are less likely to be overweight or obese than younger ones in our analysis, possibly due to increased body image awareness or BMI changes during puberty [35]. We also found that children and adolescents living in Northeast China and communities with better infrastructure are more likely to be overweight or obese. The above findings show that these key population-based factors must be considered when formulating policies to address overweight and obesity among children and adolescents and implementing interventions tailored to specific groups.

### Policy implications

The complex factors driving overweight and obesity among children and adolescents necessitate a systems-thinking approach to understanding their causes and organising the evidence required for effective action. The Government of China has attempted to tackle this issue through a multifaceted approach. It has implemented initiatives such as the Childhood and Adolescent Obesity Prevention and Control Implementation Program, Technical Guidelines on Comprehensive Public Health Prevention and Control of Overweight and Obesity in Primary and Secondary School Students, and the ‘Year of Weight Management’ campaign, among others. These efforts demonstrate the development of a broad social framework for preventing and controlling overweight and obesity among children and adolescents in China [11]. These policies and initiatives advocate for several individual, school-based, and family-based interventions; however, they still face numerous limitations.

**Figure 2:**
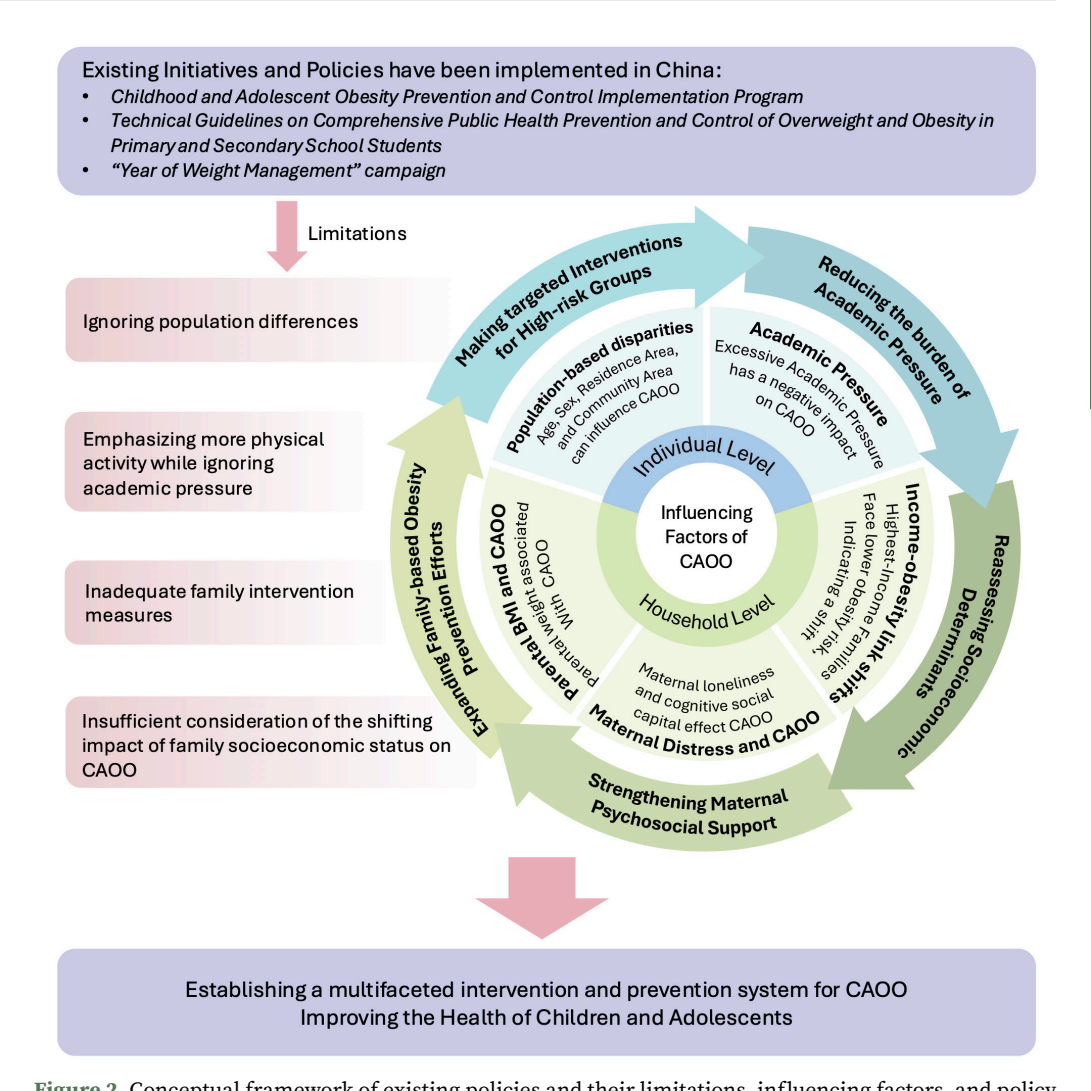
Conceptual framework of existing policies and their limitations, influencing factors, and policy implications for overweight and obesity among children and adolescents in China, which integrates findings from the CFPS 2020 with current national initiatives. The figure highlights individual and household-level factors associated with overweight and obesity among children and adolescents, identifies key limitations of existing approaches, and proposes multifaceted strategies to strengthen future prevention and intervention efforts, thus promoting children's and adolescents' health in China.

Our findings point to several priority areas in which such policy responses can be sharpened. For example, reducing academic pressure is essential in this context. Furthermore, strengthening maternal psychosocial support is critical as targeted programmes that alleviate loneliness and enhance maternal social capital may indirectly reduce overweight and obesity among children and adolescents risk through healthier family dynamics. Family-centred interventions likewise remain important, particularly in supporting healthy weight management for both parents, emphasising the role of the whole household in modelling lifestyle behaviours. Additionally, targeted interventions should be developed to address the unique needs of overweight and obesity among children and adolescents in high-risk groups. Lastly, socioeconomic determinants should be reassessed in future policymaking. Based on our findings, strengthening these areas could enhance the overweight and obesity among children and adolescents prevention efforts of China and create a more effective, holistic framework for addressing overweight and obesity among children and adolescents (**Figure 2**).

### Limitations

The main limitation of our study is the cross-sectional design, which prevented the establishment of causal relationships between variables. Because of this, the observed associations could have been affected by bias and measurement errors. Future research should prioritise longitudinal studies that allow for causal inference and explore the dynamic interplay over time. Another key limitation lies in the CFPS questionnaire design. We derived relevant measures from two separate instruments – the personal questionnaire and the children’s questionnaire – which differ in structure and content. This design limited the possibility of integrating certain variables and restricted the inclusion of important factors such as physical activity and nutritional intake. Similarly, due to the questionnaire-based design, only a broad measure of internet use was available, without information on type, duration, or content, which may have contributed to unexpected or even reversed associations. Future studies should consider expanding the scope of analysis to include a more comprehensive set of contextual factors and reassessing the role of physical activities, nutritional intake, and internet use, to obtain deeper insights into the mechanisms underlying overweight and obesity among children and adolescents.

## CONCLUSIONS

We found that overweight and obesity among children and adolescents in China is influenced by both individual factors and household-related factors, including parental weight status and maternal psychosocial well-being, as well as academic pressure and population-based disparities. Our analysis showed that traditional socioeconomic gradients were less pronounced, suggesting shifting patterns compared with findings of exsiting studies. These findings underscore the intergenerational and sociocultural pathways of overweight and obesity among children and adolescents and call for interventions that require the joint efforts of families, schools, and the government. Future research should explore the mechanisms underlying these pathways and examine how evolving social and educational contexts reshape children's and adolescents' overweight and obesity risks.

## Additional material


Online Supplementary Document
