# Supplementary material for: Individual and household determinants of overweight and obesity among children and adolescents in China
Source: J Glob Health. 2026 Apr 17;16:04096. doi: 10.7189/jogh.16.04096 (PMC13086484; doi:10.7189/jogh.16.04096)
Supplement: Online Supplementary Document [file jogh-16-04096-s001.pdf]

Supplement to: Xue M, Jiang Y, Chen M, Si L. Individual and household determinants of overweight and obesity among children and adolescents in China. J Glob Health. 2026;16:04096.

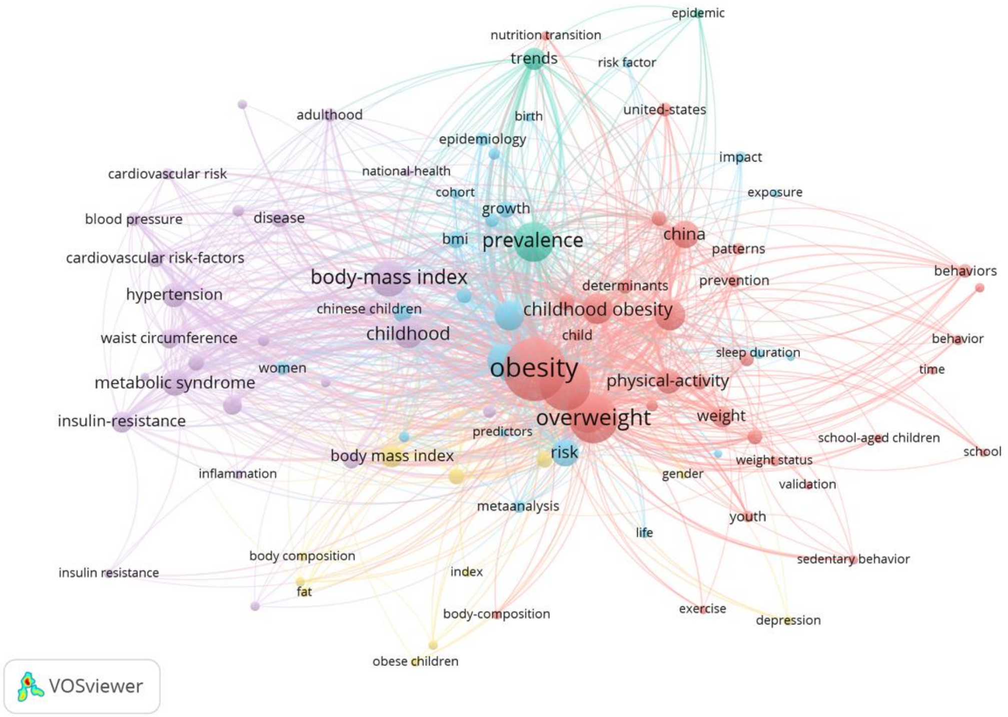

Figure S1. Keywords co-occurrence visualization map related to CAOO.

Relevant literature was retrieved from the Web of Science Core Collection using the search formula: TS=(“China” OR “Chinese”) AND TS=(“child*” OR “teen*” OR “adolescent*”) AND TS=(“overweight” OR “obesity”) AND TS=(“influence*” OR “factor*” OR “determinant*”). Keywords with a co-occurrence frequency greater than 30 were extracted for further analysis.

Table S1. Grouping criterion of dependent variables.

Age	Boys BMI, kg/m^2		Girls BMI, kg/m^2	
	Overweight	Obesity	Overweight	Obesity
7 ~	17.4	19.2	17.2	18.9
8 ~	18.1	20.3	18.1	19.9
9 ~	18.9	21.4	19.0	21.0
10 ~	19.6	22.5	20.0	22.1
11 ~	20.3	23.6	21.1	23.3
12 ~	21.0	24.7	21.9	24.5
13 ~	21.9	25.7	22.6	25.6
14 ~	22.6	26.4	23.0	26.3
15 ~	23.1	26.9	23.4	26.9
16 ~	23.5	27.4	23.7	27.4
17 ~	23.8	27.8	23.8	27.7
18	24.0	28.0	24.0	28.0

Notes: Children and adolescents were classified as overweight if their body mass index (BMI) exceeded the age- and sex-specific cutoff for overweight, and as obese if their BMI exceeded the cutoff for obesity. The criteria were developed by the Group of China Obesity Task Force and are applicable to Chinese children and adolescents aged 7–18 years.

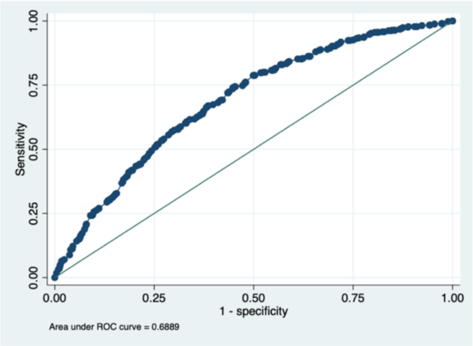

(a) ROC curve and AUC value for Overweight Model 1

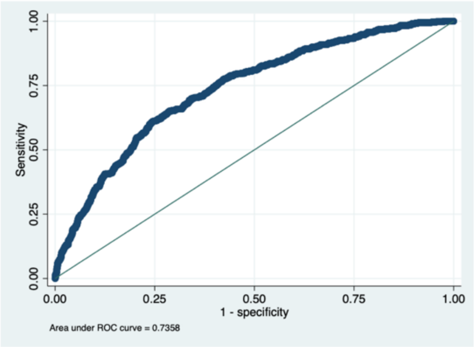

(b) ROC curve and AUC value for Overweight Model 2

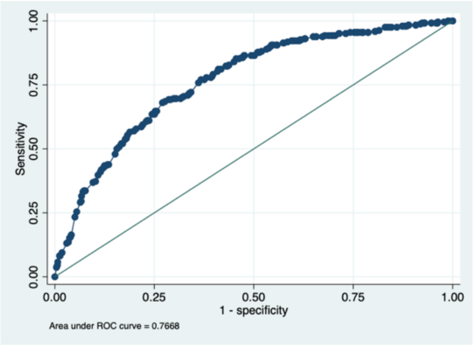

(c) ROC curve and AUC value for Obesity Model 1

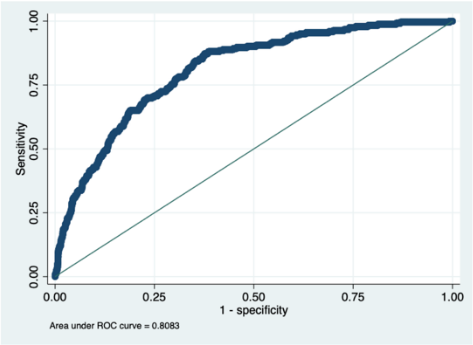

(d) ROC curve and AUC value for Obesity Model 2

Figure S2. Results of ROC (Receiver Operating Characteristic) analyses for Overweight and Obesity Models.

The AUC (Area Under the Curve) values are 0.6889 for Overweight Model 1 (a), 0.7358 for Overweight Model 2 (b), 0.7668 for Obesity Model 1 (c), and 0.8083 for Obesity Model 2 (d), indicating that all models used in this study possessed reasonable explanatory power.

Table S2. Results of Hosmer-Lemeshow Tests for Overweight and Obesity Models.

Hosmer-Lemeshow Goodness-of-fit tests indicated satisfactory model fit for all models, with Hosmer–Lemeshow p-values of .6189 for Overweight Model 1, .4196 for Overweight Model 2, .3720 for Obesity Model 1, and .3773 for Obesity Model 2.

Model	Overweight Model 1	Overweight Model 2	Obesity Model 1	Obesity Model 2
Number of observations	2,431	2,431	2,431	2,431
Number of groups	10	10	10	10
Hosmer–Lemeshow chi^2	6.25	8.14	8.66	8.6
P Value	0.6198	0.4196	0.372	0.3773

Table S3. Results of Multicollinearity Test and variance inflation factors (VIFs).

In the model including only individual factors, all VIFs were below 5, with a mean VIF of 1.16. In the model including both individual and household factors, all VIFs were also below 5, with a mean VIF of 1.18. Therefore, the models were not affected by multicollinearity.

Variable	VIF	1/VIF	Variable	VIF	1/VIF
Internet use	1.35	0.74	Maternal education level	1.68	0.60
Age	1.34	0.75	Paternal education level	1.51	0.66
Informal education	1.06	0.94	Age	1.47	0.68
Residence area	1.05	0.95	Internet use	1.43	0.70
Sex	1	1.00	Household equivalized disposable income	1.28	0.78
			Residence area	1.2	0.83
			Paternal overweight status	1.2	0.84
			Paternal obesity status	1.18	0.85
			Maternal overweight status	1.17	0.85
			Maternal obesity status	1.17	0.86
			Informal education	1.12	0.89
			Household economic region	1.1	0.91
			Mother–child age gap	1.08	0.93
			Community infrastructure status	1.07	0.94
			Paternal cognitive social capital	1.06	0.94
			Maternal structural social capital	1.06	0.95
			Maternal loneliness days in a week	1.05	0.95
			Paternal structural social capital	1.05	0.95
			Paternal cognitive social capital	1.05	0.96
			Paternal loneliness days in a week	1.04	0.96
			Parental marital status	1.03	0.97
			Sex	1.01	0.99
Mean VIF	1.16		Mean VIF	1.18	

Table S4. Results of Interaction Test for household equivalized disposable income and household economic region.

Interaction tests showed no significant effect between household equivalized disposable income and household economic region suggesting that the association between income and CAOO was consistent across economic regions.

Variables	OR (95%CI)	P	OR (95%CI)	P
	Overweight Model		Obesity Model	
**Individual level**				
Age (continuous)	0.87 (0.84, 0.91)	<0.001	0.73 (0.68, 0.78)	<0.001
Sex (ref: Female)				
Male	2.52 (2.02, 3.16)	<0.001	3.08 (2.23, 4.25)	<0.001
Residence area (ref: Rural)				
Urban	1.32 (1.05, 1.67)	0.019	1.46 (1.06, 2.02)	0.021
Informal education (ref: No)				
Yes	1.49 (1.17, 1.90)	0.001	1.59 (1.14, 2.21)	0.006
Internet Use (ref: No)				
Yes	0.78 (0.60, 1.00)	0.052	0.97 (0.69, 1.37)	0.872
**Household Level**				
Paternal overweight status (ref: No)				
Yes	1.60 (1.26, 2.03)	<0.001	1.66 (1.20, 2.31)	0.002
Paternal obesity status (ref: No)				
Yes	1.46 (1.06, 2.00)	0.020	1.73 (1.15, 2.61)	0.009
Maternal overweight status (ref: No)				
Yes	1.52 (1.20, 1.92)	0.001	1.59 (1.15, 2.20)	0.005
Maternal obesity status (ref: No)				
Yes	1.54 (1.03, 2.30)	0.037	1.51 (0.88, 2.59)	0.133
Paternal education level (ref: Illiterate)				
Below junior high school	0.83 (0.47, 1.46)	0.521	0.57 (0.26, 1.21)	0.141
Junior high school and above	0.91 (0.52, 1.61)	0.753	0.62 (0.29, 1.32)	0.213
Maternal education level (ref: Illiterate)				
Below junior high school	0.67 (0.42, 1.06)	0.089	0.57 (0.30, 1.09)	0.090
Junior high school and above	0.73 (0.46, 1.16)	0.187	0.46 (0.24, 0.87)	0.018
Parents' marital status (ref: Married)				
Other	0.51 (0.14, 1.81)	0.298	0.86 (0.18, 4.08)	0.847
Maternal-child age difference (continuous)	1.00 (0.98, 1.02)	0.884	0.97 (0.94, 1.00)	0.045
Paternal loneliness days in a week (ref: Almost never (less than a day))				
Sometimes (1-2 days)	1.06 (0.83, 1.34)	0.651	1.18 (0.86, 1.63)	0.306
Often (3-4 days)	1.03 (0.60, 1.75)	0.923	1.15 (0.55, 2.40)	0.715
Most of the time (5-7 days)	0.51 (0.23, 1.13)	0.096	0.65 (0.24, 1.76)	0.396
Maternal loneliness days in a week (ref: Almost never (less than a day))				

Sometimes (1-2 days)	1.26 (1.00, 1.59)	0.049	1.38 (1.01, 1.89)	0.043
Often (3-4 days)	1.25 (0.73, 2.14)	0.407	1.12 (0.54, 2.31)	0.765
Most of the time (5-7 days)	1.20 (0.52, 2.74)	0.669	1.46 (0.47, 4.50)	0.513
Maternal cognitive social capital (ref: High)				
Low	1.36 (1.09, 1.69)	0.006	1.47 (1.09, 1.98)	0.011
Paternal cognitive social capital (ref: High)				
Low	1.00 (0.80, 1.25)	0.981	0.90 (0.66, 1.22)	0.490
Maternal structural social capital (continuous)	1.08 (1.01, 1.15)	0.016	1.06 (0.97, 1.15)	0.180
Paternal structural social capital (continuous)	0.97 (0.91, 1.03)	0.295	1.03 (0.95, 1.13)	0.468
Household equivalized disposable income (ref: lowest25%)				
lower-middle 25%	1.16 (0.61, 2.21)	0.653	0.53 (0.22, 1.27)	0.154
upper-middle 25%	0.91 (0.48, 1.74)	0.772	0.82 (0.36, 1.85)	0.632
highest 25%	0.88 (0.48, 1.61)	0.677	0.49 (0.23, 1.07)	0.075
Community infrastructure status (ref: Poor)				
Fair	0.93 (0.74, 1.17)	0.547	0.96 (0.70, 1.33)	0.822
Good	0.60 (0.40, 0.91)	0.015	0.92 (0.55, 1.54)	0.756
Household economic region (ref: Eastern region)				
Central region	0.79 (0.41, 1.51)	0.473	0.84 (0.37, 1.89)	0.670
Western region	0.98 (0.52, 1.83)	0.943	0.54 (0.24, 1.23)	0.143
Northeastern region	1.04 (0.36, 2.94)	0.948	1.37 (0.57, 3.26)	0.481
Interaction item (ref: Eastern region*lowest 25%)				
Central region*lower-middle 25%	1.06 (0.45, 2.50)	0.897	1.85 (0.59, 5.76)	0.289
Central region*upper-middle 25%	1.18 (0.50, 2.77)	0.712	0.82 (0.27, 2.45)	0.723
Central region*highest 25%	1.15 (0.51, 2.61)	0.739	1.06 (0.37, 3.09)	0.911
Western region*lower-middle 25%	0.60 (0.26, 1.36)	0.221	1.85 (0.60, 5.70)	0.283
Western region*upper-middle 25%	1.14 (0.50, 2.58)	0.760	1.02 (0.34, 3.02)	0.977
Western region*highest 25%	0.83 (0.35, 2.00)	0.679	1.42 (0.43, 4.71)	0.562
Northeastern region*lower-middle 25%	1.07 (0.27, 4.19)	0.927	2.07 (0.48, 8.84)	0.327
Northeastern region*upper-middle 25%	2.17 (0.61, 7.66)	0.230	1.10 (0.30, 4.00)	0.884
Northeastern region*highest 25%	1.52 (0.45, 5.16)	0.505	---	---

Notes: The interaction term for Northeastern region*highest 25% income in the Obesity Model is left blank because no individuals residing in Northeastern China with the lowest 25% household equivalized disposable income were classified as obesity.

Table S5. Sensitivity analysis replacing household equivalized disposable income with household per capita disposable income for children and adolescents overweight.

Variables	OR (95%CI)	P	OR (95%CI)	P
	Model 1		Model 2	
**Individual level**				
Age (continuous)	0.89 (0.86, 0.92)	<0.001	0.88 (0.84, 0.91)	<0.001
Sex (ref: Female)				
Male	2.40 (1.93, 2.98)	<0.001	2.54 (2.03, 3.17)	<0.001
Residence area (ref: Rural)				
Urban	1.37 (1.11, 1.70)	0.003	1.32 (1.05, 1.67)	0.019
Informal education (ref: No)				
Yes	1.59 (1.27, 1.99)	<0.001	1.49 (1.17, 1.90)	0.001
Internet Use (ref: No)				
Yes	0.80 (0.63, 1.01)	0.062	0.75 (0.59, 0.97)	0.030
**Household level**				
Paternal overweight status (ref: No)				
Yes			1.63 (1.29, 2.06)	<0.001
Paternal obesity status (ref: No)				
Yes			1.43 (1.04, 1.96)	0.026
Maternal overweight status (ref: No)				
Yes			1.53 (1.21, 1.94)	<0.001
Maternal obesity status (ref: No)				
Yes			1.52 (1.02, 2.27)	0.041
Paternal education level (ref: Illiterate)				
Below junior high school			0.79 (0.45, 1.39)	0.412
Junior high school and above			0.88 (0.50, 1.53)	0.641
Maternal education level (ref: Illiterate)				
Below junior high school			0.66 (0.42, 1.05)	0.080
Junior high school and above			0.71 (0.45, 1.13)	0.152
Parents' marital status (ref: Married)				
Other			0.50 (0.14, 1.75)	0.277
Mother–child age gap (continuous)			1.00 (0.98, 1.02)	0.871
Paternal loneliness days in a week (ref: Almost never (less than a day))				
Sometimes (1–2 days)			1.04 (0.82, 1.32)	0.762
Often (3–4 days)			1.02 (0.60, 1.73)	0.948
Most of the time (5–7 days)			0.50 (0.23, 1.11)	0.091
Maternal loneliness days in a week (ref: Almost never (less than a day))				
Sometimes (1–2 days)			1.26 (1.00, 1.58)	0.051
Often (3–4 days)			1.29 (0.76, 2.20)	0.345
Most of the time (5–7 days)			1.16 (0.51, 2.65)	0.724

Maternal cognitive social capital (ref: High)		
Low	1.36 (1.09, 1.69)	0.006
Paternal cognitive social capital (ref: High)		
Low	1.00 (0.80, 1.24)	0.978
Maternal structural social capital (continuous)	1.08 (1.01, 1.15)	0.017
Paternal structural social capital (continuous)	0.96 (0.91, 1.02)	0.225
Household per capita disposable income (ref: Lowest 25%)		
Lower–middle 25%	1.01 (0.75, 1.38)	0.925
Upper–middle 25%	0.90 (0.66, 1.24)	0.527
Highest 25%	0.97 (0.69, 1.36)	0.842
Community infrastructure status (ref: Poor)		
Fair	0.94 (0.75, 1.18)	0.581
Good	0.62 (0.41, 0.93)	0.020
Household economic region (ref: Eastern region)		
Central region	0.88 (0.66, 1.16)	0.353
Western region	0.87 (0.64, 1.18)	0.371
Northeastern region	1.54 (1.04, 2.27)	0.032

Table S6. Sensitivity analysis replacing household equivalized disposable income with household per capita disposable income for children and adolescents obesity.

Variables	OR (95%CI)	P	OR (95%CI)	P
	Model 1		Model 2	
**Individual level**				
Age (continuous)	0.76 (0.72, 0.81)	<0.001	0.73 (0.69, 0.78)	<0.001
Sex (ref: Female)				
Male	2.84 (2.08, 3.87)	<0.001	3.06 (2.22, 4.22)	<0.001
Residence area (ref: Rural)				
Urban	1.25 (0.94, 1.67)	0.122	1.48 (1.08, 2.04)	0.015
Informal education (ref: No)				
Yes	1.50 (1.11, 2.03)	0.009	1.58 (1.14, 2.20)	0.006
Internet Use (ref: No)				
Yes	0.91 (0.67, 1.25)	0.578	0.96 (0.69, 1.35)	0.825
**Household level**				
Paternal overweight status (ref: No)				
Yes			1.64 (1.19, 2.27)	0.003
Paternal obesity status (ref: No)				
Yes			1.70 (1.13, 2.55)	0.011
Maternal overweight status (ref: No)				
Yes			1.63 (1.18, 2.25)	0.003
Maternal obesity status (ref: No)				
Yes			1.49 (0.87, 2.54)	0.144
Paternal education level (ref: Illiterate)				
Below junior high school			0.56 (0.26, 1.19)	0.130
Junior high school and above			0.65 (0.31, 1.37)	0.258
Maternal education level (ref: Illiterate)				
Below junior high school			0.56 (0.30, 1.07)	0.078
Junior high school and above			0.43 (0.23, 0.82)	0.010
Parents' marital status (ref: Married)				
Other			0.92 (0.20, 4.23)	0.913
Mother–child age gap (continuous)			0.97 (0.94, 1.00)	0.042
Paternal loneliness days in a week (ref: Almost never (less than a day))				
Sometimes (1–2 days)			1.14 (0.83, 1.57)	0.417
Often (3–4 days)			1.09 (0.53, 2.27)	0.812
Most of the time (5–7 days)			0.68 (0.25, 1.84)	0.446
Maternal loneliness days in a week (ref: Almost never (less than a day))				
Sometimes (1–2 days)			1.38 (1.01, 1.88)	0.042
Often (3–4 days)			1.14 (0.55, 2.33)	0.728
Most of the time (5–7 days)			1.29 (0.42, 3.96)	0.655

Maternal cognitive social capital (ref: High)		
Low	1.45 (1.08, 1.95)	0.012
Paternal cognitive social capital (ref: High)		
Low	0.91 (0.67, 1.23)	0.525
Maternal structural social capital (continuous)	1.05 (0.97, 1.14)	0.255
Paternal structural social capital (continuous)	1.04 (0.95, 1.13)	0.380
Household per capita disposable income (ref: Lowest 25%)		
Lower–middle 25%	0.97 (0.64, 1.47)	0.891
Upper–middle 25%	0.86 (0.57, 1.31)	0.485
Highest 25%	0.65 (0.40, 1.04)	0.070
Community infrastructure status (ref: Poor)		
Fair	0.96 (0.70, 1.32)	0.807
Good	0.93 (0.55, 1.55)	0.772
Household economic region (ref: Eastern region）		
Central region	0.96 (0.66, 1.40)	0.849
Western region	0.70 (0.46, 1.07)	0.098
Northeastern region	1.31 (0.76, 2.25)	0.335

Table S7. Sensitivity analysis with BMI treated as a continuous outcome.

Linear regression was estimated using BMI as a continuous variable to test the robustness of the main findings.

BMI	B (95% CI)	P
**Variables**		
**Individual level**		
Age (continuous)	0.37 (0.33, 0.42)	<0.001
Sex (ref: Female)		
Male	0.82 (0.56, 1.07)	<0.001
Residence area (ref: Rural)		
Urban	0.37 (0.09, 0.66)	0.010
Informal education (ref: No)		
Yes	0.74 (0.43, 1.06)	<0.001
Internet Use (ref: No)		
Yes	-0.14 (-0.45, 0.18)	0.406
**Household Level**		
Paternal overweight status (ref: No)		
Yes	0.76 (0.48, 1.04)	<0.001
Paternal obesity status (ref: No)		
Yes	0.80 (0.38, 1.23)	<0.001
Maternal overweight status (ref: No)		
Yes	0.88 (0.59, 1.17)	<0.001
Maternal obesity status (ref: No)		
Yes	0.85 (0.29, 1.40)	0.003
Paternal education level (ref: Illiterate)		
Below junior high school	-0.11 (-0.76, 0.54)	0.738
Junior high school and above	-0.26 (-0.91, 0.40)	0.439
Maternal education level (ref: Illiterate)		
Below junior high school	-0.47 (-1.00, 0.06)	0.083
Junior high school and above	-0.36 (-0.89, 0.17)	0.187
Parents' marital status (ref: Married)		
Other	0.07 (-1.15, 1.30)	0.905
Maternal-child age difference (continuous)	-0.02 (-0.05, 0.01)	0.157
Paternal loneliness days in a week (ref: Almost never (less than a day))		
Sometimes (1-2 days)	0.00 (-0.29, 0.30)	0.983
Often (3-4 days)	-0.06 (-0.69, 0.58)	0.864
Most of the time (5-7 days)	-0.23 (-1.05, 0.58)	0.574
Maternal loneliness days in a week (ref: Almost never (less than a day))		
Sometimes (1-2 days)	0.29 (0.00, 0.57)	0.049
Often (3-4 days)	0.15 (-0.52, 0.81)	0.667

Most of the time (5-7 days)	0.91 (-0.06, 1.88)	0.067
Maternal cognitive social capital (ref: High)		
Low	0.28 (0.01, 0.55)	0.039
Paternal cognitive social capital (ref: High)		
Low	-0.04 (-0.31, 0.23)	0.758
Maternal structural social capital (continuous)	0.09 (0.01, 0.16)	0.018
Paternal structural social capital (continuous)	-0.01 (-0.08, 0.07)	0.822
Household equivalized disposable income (ref: lowest 25%)		
lower-middle 25%	0.05 (-0.32, 0.42)	0.783
upper-middle 25%	0.10 (-0.28, 0.48)	0.609
highest 25%	-0.15 (-0.57, 0.26)	0.465
Community infrastructure status (ref: Poor)		
Fair	0.02 (-0.26, 0.30)	0.893
Good	-0.41 (-0.87, 0.05)	0.081
Household economic region (ref： Eastern region）		
Central region	0.09 (-0.25, 0.43)	0.604
Western region	-0.25 (-0.61, 0.12)	0.184
Northeastern region	0.34 (-0.18, 0.86)	0.199

Table S8 JoGH's Guidelines for Reporting Analyses of Big Data Repositories Open to the Public (GRABDROP) items

Item	Response
1. Please list all papers published by each co-author in previous three years that were based on secondary analysis of a big data repository	**Meiqiyang Xue:** None<br>**Yutong Jiang:** None<br>**Mingsheng Chen:**<br>1. Liu J, Tang Y, Zheng P, Chen M, Si L. Inequalities in health care use among patients with arthritis in China: using Andersen's Behavioral Model. Cost Effectiveness and Resource Allocation. 2024 Aug 31;22(1):61. doi: 10.1186/s12962-024-00572-x. PMID: 39217335; PMCID: PMC11366147.<br>**Lei Si:**<br>1. Liu J, Tang Y, Zheng P, Chen M, Si L. Inequalities in health care use among patients with arthritis in China: using Andersen's Behavioral Model. Cost Effectiveness and Resource Allocation. 2024 Aug 31;22(1):61. doi: 10.1186/s12962-024-00572-x. PMID: 39217335; PMCID: PMC11366147.<br>2. Chen H, Dang Q, Shi Y, et al., "The Comorbidity Burdens of Osteoarthritis and 174 Diseases, 1990–2021: A Modeling Study Based on the Global Burden of Disease Study 2021," International Journal of Rheumatic Diseases29, no. 1 (2026): e70548, https://doi.org/10.1111/1756-185x.70548.
2. Please explain the key elements of your study design and the use of the available datasets that make your study an original scientific contribution	Overweight and obesity among children and adolescents are increasingly prevalent and should be understood not only as individual health issues but also within a family context. Previous studies have primarily focused on parental physical health, with limited attention to broader household factors, particularly parental mental health and social capital.<br>This study contributes to the literature by examining a wider range of household-level determinants, including parental social capital, which remains underexplored. We used data from the China Family Panel Studies (CFPS), a nationally representative longitudinal dataset that captures detailed information on children and adolescents themselves, family members, and household environments. This enables us to simultaneously assess individual-level characteristics and household-level influences, providing a more comprehensive understanding of overweight and obesity among children and adolescents.
3. Please list all publications that addressed similar research questions in the same dataset and indicate where you cited	9 Wu YH, Moore S, Dube L. Social capital and obesity among adults: Longitudinal findings from the Montreal neighborhood networks and healthy aging panel. Prev Med. 2018;111:366–70. Medline:29197528 doi:10.1016/j.ypmed.2017.11.028<br>We cited this reference in the Background section to highlight the limited evidence on the association between parental social capital and

them in your paper	overweight/obesity among children and adolescents, as existing studies have largely focused on individuals' own social capital in relation to their weight status. In the Discussion section, we further integrated this reference with other relevant studies to interpret our findings on the relationship between parental social capital and overweight/obesity among children and adolescents.<br>10 Xie Y, Lu P. The Sampling Design of the China Family Panel Studies (CFPS). Chin J Sociol. 2015;1:471-84. Medline:29854418 doi:10.1177/2057150x15614535<br>We cited this reference in the Methods section to introduce the CFPS dataset and to describe its sampling design.
4. Please explain how you addressed multiple testing through an appropriately rigorous statistical threshold and indicate this in the methods section	Our analyses were primarily hypothesis-driven rather than exploratory; therefore, a formal multiple testing correction was not applied. However, we used a conventional significance threshold ($p < 0.05$) and interpreted the findings with caution.
5. Please declare to what extent have AI chatbots been used in developing your paper and to which parts of the paper did they contribute	AI chatbots were used only for language editing and polishing. They did not contribute to the study design, data analysis, interpretation of results, or the generation of scientific content.
